# Synbiotics and Gut-Heart Axis in Cardiometabolic Disease

**DOI:** 10.1016/j.jacbts.2024.11.008

**Published:** 2025-01-27

**Authors:** Marco Sachse, Konstantinos Stellos

**Affiliations:** a Department of Cardiovascular Surgery, University Heart and Vascular Center, University Medical Center Hamburg-Eppendorf, Hamburg, Germany; b Department of Cardiovascular Research, Medical Faculty Mannheim, Heidelberg University, Mannheim, Germany; c German Centre for Cardiovascular Research (DZHK), Partner Site Heidelberg/Mannheim, Mannheim, Germany; d Helmholtz Institute for Translational AngioCardioScience (HI-TAC), Mannheim, Germany; e Department of Cardiology, Angiology, Haemostaseology and Medical Intensive Care, University Medical Centre Mannheim, Heidelberg University, Mannheim, Germany

**Keywords:** cardiometabolic, gut-heart axis, microbiome, probiotic

**Figure:**
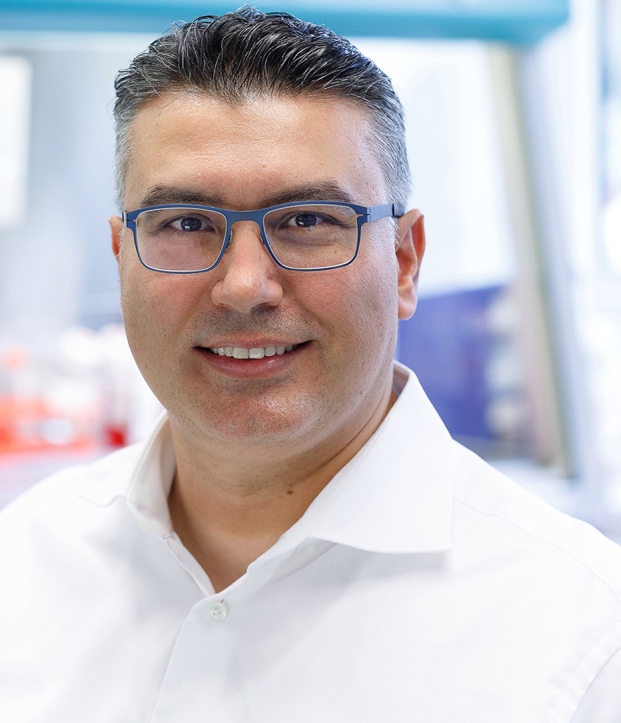

In the past 2 decades, diagnostic and pharmacologic treatments for heart failure with preserved ejection fraction (HFpEF) have significantly advanced, reflecting a deeper understanding of this complex clinical syndrome.^1^ Despite these improvements, more than 6 million adults in the United States live with chronic heart failure (HF), and the incidence and prevalence of HFpEF continue to rise, driven by factors such as aging, sedentary lifestyle, and cardiometabolic disorders (eg, arterial hypertension, abdominal obesity, diabetes mellitus, and hyperlipidemia), thus emphasizing the urgent need to identify previously unappreciated cofactors involved in the development of cardiometabolic disease with heart involvement (HFpEF).^1^ Inflammation is a major determinant of cardiovascular disease and several proinflammatory peptides, enzymes, or cytokines are associated with cardiovascular disease prognosis.^2^^,^^3^ However, the triggers of chronic inflammation in cardiometabolic disease are not well understood.

Interestingly, within the last decade, the role of the human gut microbiome in cardiovascular disease and heart failure has gained mounting evidence.^3^, ^4^, ^5^, ^6^ Among the first studies, Koeth et al^4^ reported how gut microbiota can metabolize dietary components such as L-carnitine, a nutrient found in red meat, linking microbiota to cardiovascular health and potentially foreshadowing implications for heart failure. Both murine and human studies have previously suggested the presence of an association between gut microbiota–derived metabolites and heart failure.^5^^,^^6^ Given this growing understanding of the gut microbiome's link to various diseases, therapeutic attempts, primarily through dietary modifications, have been made to shape the gut microbiome and increase the prevalence of beneficial microbes.^1^^,^^4^, ^5^, ^6^, ^7^ For instance, in mineralocorticoid excess–treated mice, supplementation with a high-fiber diet resulted in a higher prevalence of *Bacteroides acidifaciens*, which was associated with reduced cardiac fibrosis and left ventricular hypertrophy.^5^ Whether a combination therapy of prebiotics together with probiotics may be effective for the treatment of cardiometabolic disease remains to be shown (Figure 1).

**Figure 1:**
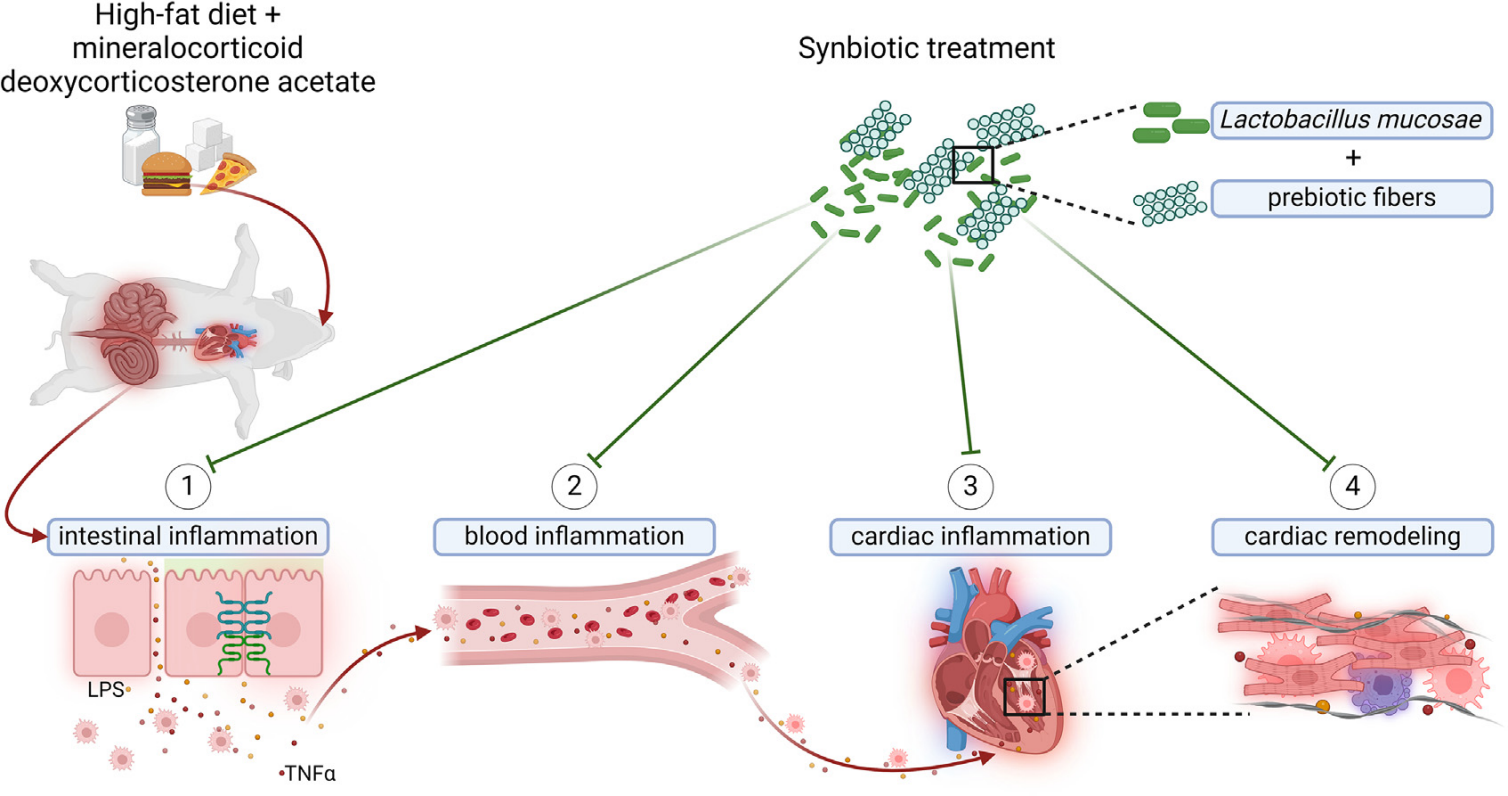
Synbiotic Treatment Improves Cardiac Health in a Porcine Cardiometabolic Disease Model A high-fat diet (HFD) combined with deoxycorticosterone acetate (DOCA) administration over 12 weeks was associated with increased inflammation in the terminal ileum. Intestinal inflammation correlated with elevated concentrations of lipopolysaccharide (LPS) and tumor necrosis factor (TNF)-α in porcine blood, alongside increased immune cell infiltration within the terminal ileum submucosa. Elevated inflammatory markers were further associated with cardiac inflammation. Higher levels of cardiac tissue inflammation were linked to cardiac remodeling, characterized by cardiomyocyte hypertrophy and increased fibrotic markers. Synbiotic treatment, comprising *Lactobacillus mucosae* and prebiotic fibers, mitigated intestinal inflammation and was associated with reduced LPS and TNF-α expression in porcine blood, as well as diminished cardiac inflammation and remodeling. However, the potential of symbiotic *Lactobacillus mucosae* treatment to mitigate blood inflammation-mediated organ damage beyond the heart remains to be investigated. Created in BioRender. Polycarpou, M. (2024) https://BioRender.com/l48s780.

In this issue of *JACC: Basic to Translational Science*, Herisson et al^8^ examine the therapeutic potential of a symbiotic treatment composed of a prebiotic fiber and a *Lactobacillus mucosa–*rich diet in a porcine model of cardiometabolic disease defined by the development of metabolic syndrome with HFpEF. It has been previously shown that a 12-week high-fat diet (HFD) increases the body weight of the experimental pigs by 10 kg, the serum lipids by 2- to 10-fold, and the insulin levels by 3-fold, alongside a notable cardiac hypertrophy and dilation of the left ventricle.^9^ In their study, Herisson et al^8^ assessed the impact of metabolic syndrome on the porcine terminal ileum by analyzing the expression of mucin 2 and key intestinal epithelial barrier proteins, including Zonulin-1 (ZO1) and Occludin (OCLN), as well as inflammatory markers within intestinal villi and crypts. The expression of mucin 2 was markedly reduced by 50% in villi and of ZO1, and OCLN by 50% both in villi as well as crypts after a 12-week HFD with deoxycorticosterone-acetate (DOCA). Additionally, intestinal TLR4, CD16-, CD11B-, and NLRP3-positive cells were elevated by 30% to 200% in the metabolic syndrome model.^8^

To confirm the presence of gut permeability in the metabolic animals, fluorescence in situ hybridization was used to label translocating bacteria from the porcine terminal ilium lumen to the crypts submucosa, and serum analysis revealed a significant elevation in gut permeability markers. After only 2 weeks of cardiometabolic diet, ZO1 levels had increased by more than 2-fold, and by 12 weeks, ZO1, lipopolysaccharide (LPS), and tumor necrosis factor (TNF)-α concentrations had risen significantly, showing nearly a 4-fold increase. In the porcine heart, the investigators demonstrated a robust activation of the NLRP3 inflammasome, upregulation of TLR4 expression, and a 20-fold increase in terminal deoxynucleotidyl transferase dUTP nick end labeling–positive cells, accompanied by increased mRNA expression of *Bax*, *BCL2*, and *Caspase 9* (*Casp9*) in the left atrium of animals with metabolic syndrome, indicating significant cardiomyocyte apoptosis.^8^ In the left ventricle of metabolic syndrome animals, similar pathologic findings were observed, including increased infiltration of CD16^+^ proinflammatory cells and enhanced NLRP3 activation. These changes were associated with left ventricular hypertrophy, as evidenced by both macroscopic and echocardiographic assessments.^8^ Taken together, the investigators provide compelling evidence of a large-scale animal model that effectively captures the intricate relationship between the gut and heart in a preclinical setting.

Building on this model, Herisson et al^8^ conducted an intervention study to evaluate the therapeutic effects of a prebiotic fiber and symbiotic Lactobacillus mucosae (SLM)–rich diet over a 12-week period. The SLM-treated animals exhibited a 30% reduction in left atrial area and a 25% reduction in left ventricular wall thickness compared to untreated metabolic syndrome animals. In terms of intestinal barrier function, the SLM treatment led to a more than 2-fold increase in mucosal layer thickness compared to the 50% reduction observed in the animals with metabolic syndrome, a layer crucial for intestinal barrier function by preventing bacteria to reach the epithelium triggering inflammation.^8^ Furthermore, a >50% reduction in CD16^+^ cells within the intestinal crypts and TNF-α serum concentrations was described by the investigators. Systemic inflammatory markers and gut permeability indicators were reduced by 30% to 50%. Similarly, the left atrium displayed 30% to 50% fewer inflammatory cells, with attenuated activation of tumor necrosis factor receptor 1 and TLR4 signaling pathways. Additionally, proapoptotic gene expression and terminal deoxynucleotidyl transferase dUTP nick end labeling positivity were decreased by 2-fold.^8^

This study from Herisson et al^8^ provides valuable insights into the effects of a *Lactobacillus*- and fiber-rich (SLM) diet in a large animal model. The investigators report that SLM treatment reduced terminal ileum submucosal and cardiac inflammation, with decreased proinflammatory immune cell infiltration and attenuation of systemic inflammation. Consistent with Herisson et al^8^, fiber-rich diets in murine models have been associated with reduced fibrosis, decreased left ventricular wall thickness, and reversal of left ventricular dilation, as observed in the deoxycorticosterone acetate-salt model.^5^ Moreover, supplementation with *Lactobacillus* following antibiotic-induced microbiome depletion post–myocardial infarction improved survival rates. This improvement was attributed to the production of short-chain fatty acids by *Lactobacillus*, which modulated the post-infarction immune response, increasing proportions of myeloid cells, macrophages, and neutrophils by 2- to 4-fold.^10^

However, in the current study from Herisson et al^8^, no alterations in the microbiome or short-chain fatty acid production were observed in response to the SLM diet. As the investigators point out, the duration of treatment required to observe microbial shifts and the full impact of microbiome-modifying diets may vary significantly between animals and humans. A 12-week study may not be sufficient to capture the complete extent of microbial and end-organ changes in humans, where disease-related structural defects accumulate over years.^1^^,^^8^ The investigators treated the animals with SLM in parallel to HFD + DOCA; however, it remains to be studied whether SLM treatment can reduce or resolve the established porcine HFpEF phenotype once metabolic syndrome is fully developed. Additionally, elucidating the full metabolome of the porcine model could reveal unidentified metabolites that contribute to the observed disease phenotype. A more detailed characterization of circulating and resident immune cells could uncover immune cell subpopulations with altered prevalence across different organ compartments.

Despite favorable outcomes in murine HF models, human studies targeting the gut microbiome have reported less success. For instance, trimethylamine N-oxide (TMAO) is strongly associated with systemic inflammation and cardiovascular diseases, including HF.^1^^,^^7^ This led to the GutHeart multicenter randomized trial involving 151 patients with a left ventricular ejection fraction below 40%. Participants were treated with either the probiotic yeast Saccharomyces boulardii or rifaximin, aimed at reducing TMAO-producing bacteria. However, the trial revealed no significant improvements in cardiac function, microbiota diversity, or systemic levels of TMAO and C-reactive protein after 3 months of treatment, raising questions about the effectiveness of targeting TMAO in patients with HF.^7^ Therefore, this clearly underscores the need for a deeper mechanistic understanding of how the gut microbiome influences cardiovascular inflammation and disease.

Several key questions remain to be addressed: Which microbiota or metabolites may influence the integrity of the intestinal epithelial barrier, and how do they mechanistically operate? How do diets affect beneficial microbes, and how do these beneficial microbes impact gut epithelial permeability and inflammation, both locally and within the heart? Do all metabolites influencing disease result from microbiome production, or do microbiome-related metabolites also affect host cells in ways that accelerate disease progression? Furthermore, are specific immune cells responsible for particular types of heart damage? The current study from Herisson et al^8^ provides valuable insights into the effects of a Lactobacillus- and fiber-rich diet in a large animal model. It remains to be shown whether the SLM diet may reduce systemic and cardiac inflammation in patients with cardiometabolic disease. Herisson et al^8^ encourage the research community to further investigate the molecular effects of microbiome-modifying treatments in this valuable human-scale disease model.

## Funding Support and Author Disclosures

Mr Sachse is supported by a physician scientist programme fellowship from the Helmholtz Institute for Translational AngioCardioScience (HI-TAC). Dr Stellos is supported by grants from the European Research Council (ERC) under the European Union's Horizon 2020 research and innovation programme (MODVASC, grant agreement No 759248), the German Research Foundation DFG (CRC1366 C07, project number 394046768), the Health+Life Science Alliance Heidelberg Mannheim GmbH and the Helmholtz Institute for Translational AngioCardioScience (HI-TAC).

## References

[1] van Ham W.B., Kessler E.L., Oerlemans M.I.F.J. (2022). Clinical phenotypes of heart failure with preserved ejection fraction to select preclinical animal models. JACC Basic Transl Science.

[2] Stamatelopoulos K., Mueller-Hennessen M., Georgiopoulos G. (2022). Cathepsin S levels and survival among patients with non–ST-segment elevation acute coronary syndromes. J Am Coll Cardiol.

[3] Gatsiou A., Tual-Chalot S., Napoli M. (2023). The RNA editor ADAR2 promotes immune cell trafficking by enhancing endothelial responses to interleukin-6 during sterile inflammation. Immunity.

[4] Koeth R.A., Wang Z., Levison B.S. (2013). Intestinal microbiota metabolism of L-carnitine, a nutrient in red meat, promotes atherosclerosis. Nat Med.

[5] Marques F.Z., Nelson E., Chu P.Y. (2017). High-fiber diet and acetate supplementation change the gut microbiota and prevent the development of hypertension and heart failure in hypertensive mice. Circulation.

[6] Wang Y.C., Koay Y.C., Pan C. (2024). Indole-3-propionic acid protects against heart failure with preserved ejection fraction. Circ Res.

[7] Awoyemi A., Mayerhofer C., Felix A.S. (2021). Rifaximin or Saccharomyces boulardii in heart failure with reduced ejection fraction: results from the randomized GutHeart trial. EBioMedicine.

[8] Herisson F.M., Cluzel G.L., Llopis-Grimalt M.A. (2025). Targeting the gut-heart axis improves cardiac remodeling in a clinical scale model of cardiometabolic syndrome. JACC Basic Transl Sci.

[9] O'Donovan A.N., Herisson F.M., Fouhy F. (2020). Gut microbiome of a porcine model of metabolic syndrome and HF-pEF. Am J Physiol Heart Circ Physiol.

[10] Tang T.W.H., Chen H.C., Chen C.Y. (2019). Loss of gut microbiota alters immune system composition and cripples postinfarction cardiac repair. Circulation.

